# The foot posture index, ankle lunge test, Beighton scale and the lower limb assessment score in healthy children: a reliability study

**DOI:** 10.1186/1757-1146-5-1

**Published:** 2012-01-09

**Authors:** Angela M Evans, Keith Rome, Lauren Peet

**Affiliations:** 1 Podiatry, AUT University, Akoranga Drive, Auckland, New Zealand; 2 Health Science, University of South Australia, North Terrace, Adelaide, Australia

## Abstract

**Background:**

Outcome measures are important when evaluating treatments and physiological progress in paediatric populations. Reliable, relevant measures of foot posture are important for such assessments to be accurate over time. The aim of the study was to assess the intra- and inter-rater reliability of common outcome measures for paediatric foot conditions.

**Methods:**

A repeated measures, same-subject design assessed the intra- and inter-rater reliability of measures of foot posture, joint hypermobility and ankle range: the Foot Posture Index (FPI-6), the ankle lunge test, the Beighton scale and the lower limb assessment scale (LLAS), used by two examiners in 30 healthy children (aged 7 to 15 years). The Oxford Ankle Foot Questionnaire (OxAFQ-C) was completed by participants and a parent, to assess the extent of foot and ankle problems.

**Results:**

The OxAFQ-C demonstrated a mean (SD) score of 6 (6) in adults and 7(5) for children, showing good agreement between parents and children, and which indicates mid-range (transient) disability. Intra-rater reliability was good for the FPI-6 (ICC = 0.93 - 0.94), ankle lunge test (ICC = 0.85-0.95), Beighton scale (ICC = 0.96-0.98) and LLAS (ICC = 0.90-0.98). Inter-rater reliability was largely good for each of the: FPI-6 (ICC = 0.79), ankle lunge test (ICC = 0.83), Beighton scale (ICC = 0.73) and LLAS (ICC = 0.78).

**Conclusion:**

The four measures investigated demonstrated adequate intra-rater and inter-rater reliability in this paediatric sample, which further justifies their use in clinical practice.

## Introduction

Outcome measures are important when evaluating effectiveness of treatment and progress towards a final goal in paediatric populations. A Cochrane systematic review published by us recently highlighted the importance of the use of reliable and validated outcome measures [1]. However, the current evidence around the use of reliable outcome measures in paediatric populations is sparse.

In the paediatric health care setting, measuring children's progress towards individual outcomes is increasingly important. Such measurements must be individual, in view of the diversity of developmental disabilities, goals, and interventions. The heterogeneity of the population often induces researchers to use generic standardised measurement tools or health-related quality of life measures; however, many are limited in terms of specificity and responsiveness to change. In contrast, in studies of homogeneous groups the sample size is often too small to detect convincing and clinically relevant differences between two treatment strategies.

Whilst flatfoot is considered to be the most common condition seen in paediatric orthopaedic clinics, it is not clear at what age children develop out of physiological flatfoot, and in the absence of obvious pathology, when and if a flatfoot is defined as pathological [2]. As a frequently reported condition it has significant implications. These are not only for the individual child, where pain or the appearance of the foot is outside normal expectations, but also for the clinician in terms of assessment and management, and the health care setting in terms of resources.

Paediatric flatfoot has been found to be associated with reduced ankle joint range of motion [3], is inversely proportional to age [4], is more prevalent in boys [5], and correlates directly with joint hypermobility [6] and being overweight/obese [7]. In complement to the clinical assessment, the Oxford Ankle Foot Questionnaire - Children can assess the extent to which the lives of children, aged 5 to 16 years, are affected by foot and ankle problems [8]. This patient-reported questionnaire takes into account the perceptions of both the child and their parent/carer into account. Usual, objective clinical assessment methods do not always capture the subjective patient perspective and may not accurately reflect how children function in their typical environments.

The reliability of clinicians' ratings is an important consideration in areas such as diagnosis and the interpretation of examination findings [9]. Reliability of clinical foot measures commonly used in paediatric foot assessments has been previously investigated in various ways and for varying purposes. For example, Macfarlane et al [10] established good intra-rater reliability for hand-held dynamometry in establishing isometric torque reference values for 154 young and healthy children for lower leg muscles. Gilmour [11] reported good intra-rater and inter-rater reliability for the measurement of the medial longitudinal arch, utilising the arch index calculated from footprints, in 272 children. This same study also established good intra-rater and inter-rater reliability for the measure of navicular height from the floor in standing subjects. Navicular height (NH), the Foot Posture Index (FPI), resting calcaneal stance position (RCSP), neutral calcaneal stance position (NCSP), navicular drop (ND) were examined in young children (4 to 6 years) and adolescents (8 to 15 years) in an intra-rater and inter-rater reliability study [12]. This study found differences in the reliability of the measures between the two age groups of children, with much lower inter-rater reliability of measures in the younger children. From this study came the notion that young children require a different approach to foot posture assessment, from which the more recent paediatric flat foot proforma has evolved, and for which adequate inter-rater reliability has been found [13].

Morrison found good intra-rater reliability for ND in 13 children [14] and also found good inter-rater reliability for the FPI in children aged 5 to 16 years [15]. The reliability of measures of ankle range has been sparsely examined in healthy children [16]. Bennell et al have established the reliability of the weight-bearing ankle lunge test in adults [17], and whilst having used the same to examine ankle motion in ballet dancers (aged 8 to 11 years), did not examine the reliability of this measure in this younger sample [18,19]. Measures of joint hypermobility (the Beighton scale and the lower limb assessment score) have demonstrated good inter-rater reliability in adults and children respectively [20-23].

Whilst there have been some recent attempts to examine aspects of paediatric foot posture and joint range in children, the results are based upon differing subject samples and methodologies. Hence, the aim of this study was to examine the intra and inter-rater reliability of clinical measures of foot posture, joint hypermobility and ankle joint range in a test-retest analysis of the same sample of healthy children.

## Methods

### Participants

Thirty children were recruited as a convenience sample from the Auckland University of Technology podiatry clinic and from staff associated with this clinic. All participants were healthy, asymptomatic children, aged between 7 and 15 years of age. The institutional ethics committee approved the study and parents/guardians provided written informed consent.

### Measurement

Demographic and participant characteristic information including age, gender, height, weight, body mass index was determined for each child at baseline. In addition, the parent and child versions of the Oxford Ankle Foot Questionnaire - Children (OxAFQ-C) were completed as the initial stage of data collection. The OxAFQ-C is a validated instrument used to assess the disability associated with foot and ankle problems in children aged from 5 to 16 years. Scores from the questionnaire can be calculated in three domains of children's lives: physical, school and play, and emotional. The questionnaire is appropriate for children with a range of conditions and can provide clinically useful information to supplement other assessment methods [8].

Four other foot and ankle musculoskeletal measurement instruments or tools were used in this study (determining the reliability of each was the primary aim of this study). These instruments included; the Foot Posture Index (FPI-6) [24], the Beighton scale [23] and the lower limb assessment score (LLAS) [20]. The fourth test, the ankle lunge test, utilised a digital read-out inclinometer to record lower leg angulations. The two examiners were both podiatrists. One examiner had 20 years clinical experience (rater 1), whilst the other examiner was newly graduated podiatrist with 1 year of clinical experience (rater 2).

### Procedure

The participating child and their parent/guardian each completed their respective versions of the OxAFQ-C (15 questions). The maximum score for these questionnaires is 15, with lower scores indicative of more severe disability [8]. Each child was then independently assessed twice by each examiner, for each of the FPI-6, Beighton scale, LLAS and ankle lunge test. At least two hours separated the assessment periods. All data collection occurred over three consecutive days.

The FPI-6 was evaluated with each child standing and using the original protocol [24]. FPI-6 values ranged from -2 to +2 for each of the six criteria and from -12 to +12 for the total score, indicative of position of each foot along the supinated (a -ve score) to pronated (a +ve score) continuum of foot posture.

The Beighton scale [22,23] was rated to ascertain the presence of joint hypermobility at the wrist, fifth metacarpal phalangeal joint, elbow, knee (all bilateral and non-weight-bearing) and the lumbo-sacral spine (forward flexion, in stance). The Beighton scale yields a score from a 9-point rating, whereby the usual arbitrary cut-off of 5/9 or greater indicates joint hypermobility [23].

The LLAS [20] was assessed to gauge joint hypermobility of the lower limb (hip, knee, ankle, subtalar, midtarsal and first metatarsophalangeal joint). The subtalar joint assessment only involved weight-bearing evaluation. The LLAS yields a 12-point score/side, and by convention the total (24 point) score is halved to deliver a final score out of 12 (with arbitrary cut-off of 7/12 or greater indicative of joint hypermobility) [20].

The ankle lunge test was performed using the method described by Bennell et al [17] and adapted by Irving et al [25]. This method incorporates an inclinometer (Smart Tool™) held on the anterior surface of the tibia, which is used to measure the participant's lunge angle. As previous works [17,19] have shown the lunge test to return symmetrical results and reliability, only the left-side lunge angle was measured for the purposes of this study.

### Data management and statistical analysis

Following data collection, all data were entered and statistical analysis was conducted using SPSS Version 17 for Windows (SPSS, Inc., Chicago, IL, USA). Mean (SD) and n (%) were used to explore the demographic and participant characteristic data.

Reliability analysis was assessed by calculating the intraclass correlations (ICCs) for each of the FPI-6, Beighton scale, LLAS and ankle lunge test (ICC (2,k) absolute agreement). ICCs across the same-subject repeated measures trials were calculated for each of the two examiners (intra-rater) and between the two examiners (inter-rater). Interpretation of the ICCs was conducted in accordance with Portney and Watkins [26], whereby values > 0.75 indicate good reliability, values ranging from 0.50 to 0.75 indicate moderate reliability and values < 0.50 imply poor reliability. We also used standard error of measurement (SEM) statistics. The SEM is expressed in the actual unit of the measurement, which is very useful: the smaller the SEM, the more reliable the results [27].

## Results

Descriptive information for the participants is presented in Table 1. Females constituted two-thirds (n = 20, 65%) of the sample and the majority of the children were of New Zealand/European ethnicity (n = 27, 90%). The OxAFQ-C demonstrated a mean (SD) score of 6 (6) in adults and 7 (5) for children, showing good agreement between parents and children, and indicating mid-range disability (which may include transient injuries) within this small, convenience sample.

**Table 1:** Participant characteristics (n = 30)

Variable	Value
Age, years, mean (SD), range	10.6 (2.3), 7.0 - 15.0
Females, n (%)	20 (65%)
Weight, kg, mean (SD)	38.7 (12.4)
Height, m, mean (SD)	1.39 (29.5)
BMI, kg/m2, mean (SD)	18.2 (3.4)
Ethnicity, n (%)	Caucasian, 27 (90%)
	Maori, 1 (3%)
	Asian, 2 (7%)
OxAFQ-C (Parent), mean (SD)	6 (6)
OxAFQ-C (Child), mean (SD)	7 (5)

Based upon the Portney and Watkins criteria [26], we found good intra-rater reliability for the FPI-6 (ICC = 0.93 - 0.94), Lunge test (ICC = 0.85-0.95), Beighton scale (ICC = 0.96-0.98) and LLAS (ICC = 0.90-0.98). The SEM was found to be low across both raters ranging between 0.4 to 2.7. These results are detailed in Table 2.

**Table 2:** Intra-rater reliability results for each examiner, across both testing periods (n = 30).

Variable	Trial 1 Mean (SD)	Trial 2 Mean (SD)	ICC (95%I)	SEM
**Rater 1**				
Foot Posture Index, mean (SD)	4 (2)	4 (3)	0.93 (0.86-0.97)	0.7
Lunge test, mm, mean (SD)	41 (7)	44 (7)	0.85 (0.67-0.93)	2.7
Beighton scale, mean (SD)	3 (2)	3 (3)	0.98 (0.95-0.99)	0.4
Lower limb assessment score, mean (SD)	8 (6)	9 (6)	0.98 (0.96-0.99)	0.9

**Rater 2**				
Foot Posture Index, mean (SD)	6 (3)	6 (3)	0.94 (0.87-0.97)	0.7
Lunge Test, mean (SD)	44 (6)	46 (8)	0.95 (0.89-0.98)	1.6
Beighton scale, mean (SD)	2 (2)	2 (2)	0.96 (0.92-0.98)	0.4
Lower limb assessment score, mean (SD)	11 (5)	11 (4)	0.90 (0.79-0.95)	1.4

Inter-rater reliability (repeated measure between rater) was largely good for each of the measures as follows: FPI-6 (ICC = 0.79), Lunge test (ICC = 0.83), Beighton scale (ICC = 0.73) and LLAS (ICC = 0.78) (Tables 3 and 4). The SEM for all measures ranged between 1.1 to 3.4.

**Table 3:** Inter-rater reliability for each measure and SEM for each of the repeated trials (n = 30)

Variable	Trial 1 ICC (95% CI)	SEM	Trial 2 ICC (95% CI)	SEM
Foot Posture Index	0.71 (0.38-0.86)	1.4	0.86 (0.72-0.94)	1.1
Lunge test	0.87 (0.72-0.94)	2.3	0.79 (0.56-0.90)	3.4
Beighton scale	0.72 (0.42-0.87)	1.1	0.74 (0.46-0.88)	1.3
Lower limb assessment score	0.84 (0.67-0.93)	2.2	0.72 (0.41-0.87)	2.7

**Table 4:** Inter-rater reliability: mean inter-rater ICC's (95% CI's) and SEM across both testing trials (n = 30)

Variable	ICC (95% CI)	SEM
Foot Posture Index	0.79 (0.38-0.94)	1.3
Lunge test	0.83 (0.56-0.94)	2.9
Beighton scale	0.73 (0.42-0.88)	1.2
Lower limb assessment score	0.78 (0.41-0.93)	2.5

## Discussion

The OxAFQ-C raw domain scores demonstrated good agreement between parents and children (Table 1). In this study's small convenience sample, little more can be inferred from these findings. The OxAFQ-C for children was developed as a site-specific (ankle/foot) instrument to provide an inexpensive and expedient method for assessing health status and evaluating outcomes from the child's perspective, aged between 5 and 16 years [8]. This objective measure should be regularly used to assess the extent to which children are affected by foot and ankle problems.

The examiners displayed largely good intra-rater and inter-rater reliability for the FPI-6, Lunge test, the Beighton scale and the LLAS when applied to the sample population of children with a mean age 10.6 years. Intra-rater reliability results returned very good intraclass correlation results and small SEM for each measure. Rater 1 was the more experienced of the two raters and returned lower FPI-6 scores and also lower LLAS scores, indicating that experience and clinical exposure modulates assessment of flat feet and joint hypermobility within the lower limb.

Inter-rater reliability results, as categorised by the Portney and Watkins levels [26], were good for the FPI-6, the lunge test and the LLAS. The Beighton score was only slightly short of this cut-off level, and being upper limb dominant may be a less familiar clinical tool for podiatrists, especially podiatry students. Clinicians can feel confident in using the FPI-6, the lunge test and either hypermobility evaluation tool in a busy clinical setting.

This study confirms the reliability of the FPI-6 [12,15,24] and the LLAS [20] in the paediatric setting. Whilst widely used as an expedient measure of global joint hypermobility, the Beighton scale has not previously been examined for its reliability in children. Previous studies in adults have found the Beighton scale to yield good inter-rater reliability [21-23]. The lunge test [17,28] has been demonstrated to be a reliable clinical tool for ankle joint range assessment in adults [17,25], but has not been tested for reliability in the paediatric population until now.

Given the known relationships between foot posture and ankle range, ankle range and hypermobility, and foot posture and hypermobility it is pertinent to have identified the most useful measures for clinical assessment of these parameters. Often used in concert, the clinician and researcher can assuredly use the FPI-6, the lunge test, the Beighton scale and/or the LLAS for both baseline and monitoring purposes.

The LLAS has distinct advantages for use in the podiatry setting as it evaluates hypermobility in the lower limb and foot very specifically. The LLAS does take longer to administer than the briefer, and more global Beighton scale, but yields far greater information distal to the hips. The Beighton scale is a very quick and slightly coarser filter for hypermobility screening, and in one author's (AE) experience, is usefully used prior to the more specific LLAS.

This study had limitations, as the sample included children with a mean age of 10.6 (2.3) years were assessed for the purpose of assessing the reliability of the four clinical tools. Caution must be advised if using these measures in ages that are significantly less or more than 10 years, and especially in younger children, for whom very different results with clinical foot measures have been previously found [12]. The clearly disparate examiner experience appears to affect results and must be noted in the assessment of both joint hypermobility and foot posture, where less experience may over-estimate extent.

Future research directions include the establishment of normative reference values across age groups for each of the four measures: the FPI-6, the lunge test, the Beighton scale and the LLAS. Such values already exist for the FPI-6 [4], so the assignation of normal values for the other three measures for healthy children and specific disease groups (e.g. cerebral palsy, Down's syndrome) would greatly assist both clinicians and research teams.

## Conclusion

The present study has, for the first time, found that the four measures of the FPI-6, the lunge test, the Beighton scale and LLAS, demonstrate adequate intra-rater and inter-rater reliability in a paediatric sample. These findings indicate that all of these measures are useful in both clinical settings and research protocols that address the paediatric foot.

## Competing interests

The authors declare that they have no competing interests.

## Authors' contributions

AE and KR contributed to conception and design of the study. LP and AE acquired the data. KR and AE analysed and interpreted the data. All authors were involved in drafting the manuscript and have given final approval of the version to be published.
